# Revisiting the Oral–Gut Axis: Microbial Symbiosis, Dysbiosis, and Bidirectional Links Between Periodontitis and Inflammatory Bowel Disease

**DOI:** 10.3390/microorganisms14071551

**Published:** 2026-07-15

**Authors:** Federica Di Gregorio, Alessandro Polizzi, Giorgia Maria Marmo, Angela Angjelova, Elena Jovanova, Roberto Campagna, Marco Mascitti, Gaetano Isola

**Affiliations:** 1 Unit of Periodontology, Department of General Surgery and Surgical-Medical Specialties, School of Dentistry, University of Catania, 95123 Catania, Italyalessandro.polizzi@phd.unict.it (A.P.);; 2 International Research Center on Periodontal and Systemic Health “PerioHealth”, University of Catania, 95123 Catania, Italy; 3 Department for the Promotion of Human Science and Quality of Life, San Raffaele Roma University, 00166 Rome, Italy; 4 Department of Clinical Specialistic and Dental Sciences, Marche Polytechnic University, 60126 Ancona, Italy

**Keywords:** dysbiosis, periodontitis, saliva, inflammatory bowel disease, oral microbiota, gut microbiota, Crohn’s disease, ulcerative colitis

## Abstract

Background: Recent scientific evidence indicates that the oral–gut axis represents a critical interface in host–microbiota interactions, carrying profound implications for both periodontal and gastrointestinal diseases. This scoping review aims to evaluate the reciprocal influence between periodontitis and inflammatory bowel disease (IBD). Specifically, the underlying mechanisms of microbial translocation, immune interaction, and metabolite-mediated signaling linking the oral microbiota to gut microbial ecology are critically evaluated. Methods: Studies were selected from the PubMed, Web of Science, and Scopus databases up to May 2026. Eligible criteria included in vivo studies written in English and conducted within the last 10 years, whereas human studies involving participants under 18 years of age were excluded. The included studies analyze the effects of oral and gut dysbiosis on the opposing district. Through the database search, 2094 records were identified, and 34 articles were selected based on the eligibility criteria. Results: The included studies demonstrate the interconnection between the oral and gut microbiota. The included studies reveal that predominant oral taxa, specifically *Porphyromonas gingivalis*, *Fusobacterium nucleatum*, and *Aggregatibacter actinomycetemcomitans*, alter intestinal microbial composition. In particular, *P. gingivalis* colonizes the gut, exacerbating both oral and intestinal inflammation by stimulating pro-inflammatory cytokine expression via Th17 cell activation. Finally, salivary microbial composition appears to be associated with the presence and status of IBD. Conclusions: Understanding these interconnected microbial ecosystems provides valuable insights that may support the future development of integrated diagnostic and therapeutic strategies for patients suffering from periodontitis and IBD. Further large-scale studies with longer follow-up periods are required to standardize potential salivary markers and multidisciplinary therapeutic protocols for the management of periodontitis and IBD.

## 1. Introduction

The human organism hosts trillions of commensal microorganisms. Bacterial species colonize various body districts, including the oral cavity, the gut, and the skin. Due to the differing characteristics of each environment, the respective microbiota exhibit distinct organization and microbial populations [1]. The oral microbiota is one of the most diverse ecosystems in our organism, second only to the gut microbiota. It consists of approximately 770 bacterial species, along with archaea, fungi, viruses, helminths, and protists [2]. The presence of these microorganisms within the oral cavity is essential for the execution of normal physiological functions in this district. They play a crucial role in maintaining oral and systemic homeostasis, which is achieved through dynamic interaction with the host via molecular, immunological, and microbial cross-talk [3]. Among the bacteria most physiologically prevalent in the oral cavity are *Firmicutes*, especially *Streptococcus*. These are capable of adhering to the dental surface due to the production of polysaccharides and surface proteins. These macromolecules determine the formation of a thin biofilm that protects against colonization by certain pathogenic germs [4]. If this plaque, produced by commensals, accumulates in larger quantities due to an inadequate lifestyle (e.g., poor oral hygiene and cigarette smoking), it becomes a predisposing factor for colonization by pathobionts (opportunistic pathogens) [5]. The preponderance of pathogenic germs within this context leads to the establishment of dysbiosis. Dysbiosis can be caused by oral pathologies such as caries and periodontitis [6,7].

Oral cavity bacterial species may be increased especially during periodontitis [8,9,10], a multifactorial disease of the gum tissue initiated usually by pathogenic oral biofilm that affects both teeth [10,11] and implants [12,13]. Periodontitis is a chronic inflammatory pathology characterized by the irreversible destruction of the tooth-supporting tissue, which consists of cementum, periodontal ligament, and alveolar bone [14]. The development of this pathology is determined by the predominant presence of periodontopathogens responsible for the onset of dysbiosis. The microorganisms highlighted in the literature as principally involved are *Porphyromonas gingivalis*, *Parvimonas micra*, *Fusobacterium nucleatum*, *Prevotella nigrescens*, *Prevotella intermedia*, *Campylobacter rectus*, *Tannerella forsythia*, *Aggregatibacter actinomycetemcomitans*, and *Treponema denticola* [15].

**Figure 1:**
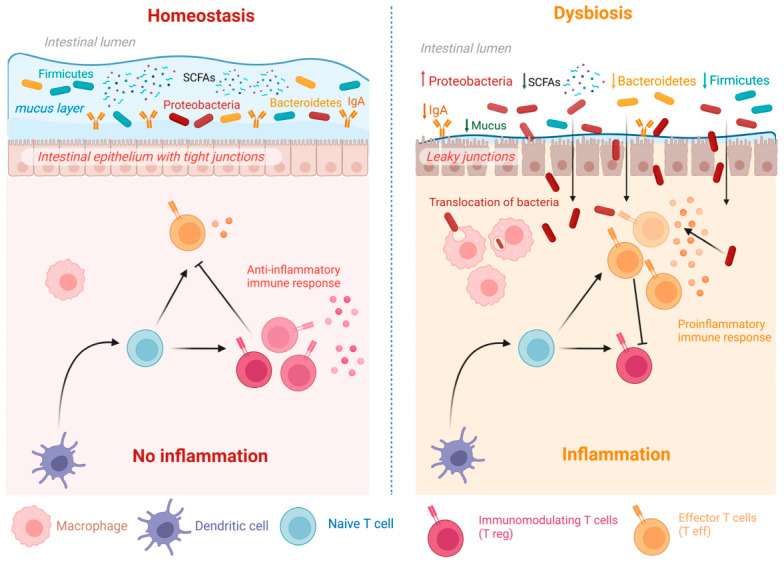
Homeostasis versus dysbiosis. Under eubiotic conditions, a homeostatic balance exists between the host immune system and the gut microbiota. Under dysbiotic conditions, a reduction in beneficial short-chain fatty acid (SCFA)-producing bacteria and the activation of the host inflammatory response occur, disrupting intestinal homeostasis. This cascade leads to the impairment of the intestinal epithelial barrier, thereby permitting the translocation and ectopic colonization of oral pathogenic bacteria. Reprinted as an exact copy with permission from Elzayat et al. [20] under Creative Commons License.

Analogously to the oral cavity, the gut is also colonised by a highly complex microbiota comprising thousands of bacterial species. The gut microbiota is the most diverse ecosystem in the human body, consisting of trillions of microorganisms. What allows for the presence of this vast variety of microbial populations, similarly to the oral cavity, are its unique characteristics, which include a nutrient-rich environment and areas with differing oxygen gradients. The latter is one of the factors determining the great variability of the microbial populations colonising the various intestinal tracts [4,16]. In healthy individuals, the gut bacterial flora is principally composed of *Proteobacteria*, *Bacteroidetes*, *Actinobacteria*, and *Firmicutes*, specifically accounting for 90% of the total population [17]. Anaerobic *Firmicutes* in the colon are capable of fermenting complex carbohydrates into short-chain fatty acids (SCFAs). In patients suffering from IBD (Inflammatory Bowel Disease), there is a reduction in the stability and variability of the gut microbiota, especially regarding Firmicutes and, in general, SCFA-producing bacteria [18] (dysbiosis; Figure 1). Conversely, an increase in *Clostridium bolteae*, *Escherichia coli*, and *Ruminococcus gnavus* is observed [18,19].

Under physiological conditions, the interaction between gut bacteria and oral bacteria is highly limited. In the event of dysbiosis, ectopic colonization by oral microorganisms can occur in other districts of the gastrointestinal tract, leading to the establishment of pathological conditions. Oral bacteria are thought to induce the alteration of the normal gut microbiota, resulting in an increase in pro-inflammatory cytokines and a reduction in SCFA-producing bacteria [4]. Currently, two potential pathways are hypothesized for gut colonization by oral pathogens: enteral and hematogenous dissemination [16,18]. Under conditions of gut dysbiosis, the permeability of the intestinal epithelial barrier increases. This alteration would allow oral bacteria ingested during physiological digestion to pass through the damaged barrier and colonize the gut [4].

The interaction between the oral microbiota and the gut microbiota is bidirectional. Indeed, in subjects affected by IBD, variations in the oral microbial composition have been highlighted, with the presence of abundant levels of the candidate phylum *TM7* (*Saccharibacteria*) and the genus *Veillonella* compared with healthy subjects [21]. Furthermore, the presence of a distinct oral microbial profile has been observed between subjects affected by periodontitis alone and subjects affected by both periodontitis and IBD [18].

Given the close correlation between the oral and gut microbiota, our scoping review aims to critically analyze the underlying mechanisms of microbial translocation, immune interaction, and metabolite-mediated signaling linking the oral microbiota to gut microbial ecology. Emphasis is placed on the salivary microbiota, potential inflammatory and metabolic markers, and their possible utilization as a non-invasive test for the screening and monitoring of IBD.

## 2. Materials and Methods

### 2.1. Study Design

This scoping review was structured following the PRISMA-ScR (Preferred Reporting Items for Systematic reviews and Meta-Analyses extension for Scoping Reviews) checklist [22], with the objective of selecting studies consistent with the research question, defining exclusion and inclusion criteria, filtering data, and highlighting results.

### 2.2. Objective and PCC Framework

This scoping review plans to investigate the existing evidence regarding the bidirectional correlation between periodontitis and IBD. This review was conducted following the Population–Concept–Context (PCC) reference framework:

○ Population (P): Human subjects affected by periodontitis, human subjects affected by IBD, animal models of periodontitis, animal models of IBD.
○ Concept (C): Periodontitis, IBD, oral dysbiosis, gut dysbiosis, immune crosstalk, and metabolite-mediated signaling.
○ Context (C): Clinical and animal experimental studies evaluating the bidirectional association between periodontitis and IBD.

### 2.3. Eligibility Criteria

The inclusion criteria considered in this review are: (1) studies analyzing the relationship between periodontitis and IBD; (2) studies on the oral or gut microbiome; (3) studies conducted within the last 10 years; (4) studies written in English; (5) in vivo animal models evaluating the oral–gut axis; (6) clinical studies conducted on human subject aged ≥ 18 years.

The following were excluded: (1) reviews; (2) case reports; (3) case series; (4) opinion pieces; (5) theses; (6) in vitro studies; (7) conference reports; (8) subjects affected by other pathologies; (9) studies that examine viruses.

Among these, in vitro cell culture models were deliberately excluded because they cannot replicate the complex, systemic multi-organ crosstalk and integrated immune responses inherent to the live bidirectional oral–gut axis. Furthermore, from a methodological standpoint, omitting in vitro data was essential to avoid excessive methodological heterogeneity, thereby ensuring a coherent and focused mapping of the available clinical and translational evidence.

### 2.4. Information Sources and Search Strategy

The bibliographic search was performed in May 2026 using Scopus, PubMed, and Web of Science as electronic scientific databases. The search strategy was designed to identify all articles exploring the oral–gut axis, with a focus on microbial translocation, microbiome alteration, immune crosstalk, and molecular pathways in subjects affected by periodontitis and/or IBD. Combinations of MeSH terms were utilized using Boolean operators and free-text keywords. Manually retrieved articles were also included.

### 2.5. Study Selection

**Figure 2:**
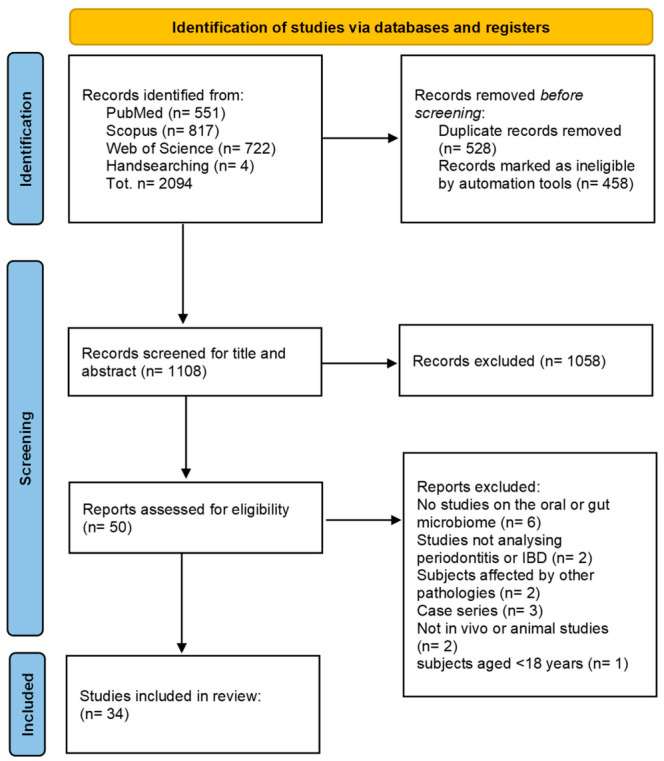
PRISMA-ScR flow diagram of the articles’ identification.

After removing duplicate articles, two independent authors screened all studies and selected the relevant ones. The first screening of the articles was conducted by evaluating titles and abstracts. The selected articles were then assessed in detail in their full-text version based on the inclusion and exclusion criteria. In the event of discrepancies between the two authors following the comparison of the final results, a third reviewer was consulted. The screening and selection process was executed following the PRISMA 2020 workflow and is illustrated in the PRISMA-ScR flow diagram (Figure 2).

## 3. Results

The search allowed for the identification of a total of 2094 records. After removing duplicates and evaluating titles and abstracts based on the inclusion and exclusion criteria, a total of 50 articles were selected. These articles were analyzed by evaluating the full text. At the end of the selection process, 34 records were identified: 2 longitudinal observational studies, 9 cross-sectional observational clinical studies, 3 prospective clinical studies, 1 multicenter observational study, 1 combined clinical and in vitro experimental study, 2 observational case–control studies, 2 observational clinical studies, 1 experimental animal study with human microbiota analysis and 13 experimental studies on animal models.

### Summary of Findings

The selected studies highlight the correlation between the oral microbiota and the gut microbiota. Eight experimental in vivo animal studies analyze the effects of periodontal pathogens on the oral microbiota [23,24,25,26,27,28,29,30]. Specifically, six studies show how *P. gingivalis* is able to alter the gut microbial composition, leading to or worsening dysbiosis [23,24,25,26,27,28]. The alteration involves not only the bacterial but also the fungal composition [25]. The presence of this pathogen in the gut and the consequent dysbiosis lead to an alteration of the host–microbiota homeostasis with inflammatory, metabolic, and systemic consequences. Dysbiosis results in the replacement of beneficial microorganisms that produce SCFAs with pathogenic ones, with a consequent reduction in lipid and amino acid metabolic pathways [23,25,26]. Furthermore, four of the previously cited studies highlighted the ability of *P. gingivalis* to increase intestinal inflammation by increasing the expression of pro-inflammatory cytokines [24,26,27,28,30] and activating Th17 cells [27,28], and one study demonstrated an alteration of the intestinal barrier [24]. Other periodontopathogens (*P. intermedia* [24], *F. nucleatum* [24,30] and *A. actinomycetemcomitans* [29,31]) are also capable of exacerbating colitis through an increased inflammatory response [24,29,30,31], the alteration of the microbiota [29,30,31] and the alteration of the intestinal barrier [24,31]. Intestinal dysregulation is greater in cases of polymicrobial intestinal translocation [29]. Evidence of the synergistic effect of oral pathogens on the gut is highlighted by two studies on murine models analyzing the effects of saliva on the gut [31,32]. In cases of oral and/or intestinal dysbiosis, the bacteria present in saliva are able to induce intestinal microbial translocation, with an increased inflammatory response and alteration of the integrity of the intestinal barrier [31,32]. Five studies conducted on subjects affected by periodontitis confirm what has been stated so far, highlighting the presence of dysbiosis [33,34,35,36,37], with alteration of the inflammatory response [34,35,36], and a reduction in intestinal metabolism of amino acids, sugars, and SCFAs [36]. In contrast, one study highlights only minimal microbial translocation with alteration of intestinal metabolic activity [38]. This study also highlights a minimal effect of non-surgical periodontal therapy on the gut microbiota [38]. These results stand in contrast to what was shown by other studies, which instead highlighted a partial significant improvement not only in the microbial balance [35,36,37]. The difference in the results could be due to a shorter waiting time before re-evaluation in the first study [38] compared with the others [35,36,37]. Studies on murine models have highlighted that treatments with postbiotics [39,40], probiotics [30] or antibiotics [41] are also able to improve intestinal dysbiosis [30,39,40,41], reduce inflammation [30,39,40,41], and improve intestinal integrity [41] altered due to periodontitis. Two observational cross-sectional studies and one observational case–control study highlight bacterial translocation from the oral cavity to the gut and the consequent ability to worsen dysbiosis in the colon of subjects affected by IBD [42,43,44,45] with a reduction in bacteria involved in butyrate and indole metabolism. A study conducted on colonic resections from subjects affected by IBD demonstrated the presence of *Fusobacteria*, *Proteobacteria*, and *Firmicutes* in colonic segments with reduced motility compared with adjacent healthy segments [42]. Furthermore, the analysis of the gut microbiome and, specifically, the identification of specific oral bacteria could predict the persistence of symptoms in subjects affected by Crohn’s disease [45].

The relationship between oral and intestinal microbiota is a bidirectional relationship. One study conducted on a murine model highlighted how colitis is able to alter the oral microbiota with epithelial hyperplasia, probably caused by the reduction in *Streptococcus* [46]. Nine studies showed that oral dysbiosis is present in subjects affected by IBD [31,43,44,47,48,49,50,51,52,53] with an enrichment of anaerobic genera (*Prevotella* spp., *Veillonella* spp.) [44,51] and pro-inflammatory taxa [48,49], with a reduction in alpha diversity [52] and a reduction in *Streptococcus*, *Haemophilus*, and *Neisseria* [44,54]. From these studies, an increase in bacterial metabolism of butyrate [49], arginine, and isoprene [43] was also detected. All these factors result in an increase in inflammatory parameters (Papillary Bleeding Index, PBI and active Matrix Metalloproteinase, aMMP8) and greater tissue destruction (increased Clinical Attachment Level, CAL) [47]. The studies also revealed a different alteration of the oral microbiota between subjects affected by Crohn’s disease and Ulcerative Colitis [47,50,52]. Two studies highlighted a variation in the salivary microbiota in subjects undergoing therapy with biologic drugs [54,55]. One observational cross-sectional study highlighted a reduction in species richness with an increase in *Prevotella*, *Campylobacter*, and *Fusobacterium* in subjects undergoing therapy with biologic drugs [54], while one observational study highlighted an increase in beneficial taxa [55]. This discrepancy in the results could be due to the lack of specificity in the choice of drug in the first study. In fact, the analysis of the microbiota was performed in subjects taking different biologic drugs. Four studies conducted on the saliva of subjects affected by IBD suggest the possibility of using salivary bacteria as specific markers for identification [44,50,53,56], monitoring [56], and the evaluation of treatment response [55] in subjects affected by IBD. Furthermore, the salivary microbiota might be able to distinguish Ulcerative Colitis from Crohn’s disease [50,56] (Table 1).

## 4. Discussion

This scoping review aims to evaluate how dysbiosis of the oral cavity and gut can not only determine the onset and progression of pathologies within their respective areas but can also act by influencing the progression and severity of diseases in the opposing district. Specifically, our article aims to analyze recent advancements regarding the influence of the oral cavity microbiota on gastrointestinal tract pathologies such as IBD, and of the gut microbiota on periodontitis. Greater emphasis is placed on the possibility of exploiting these recent discoveries as therapeutic and diagnostic strategies for better management of IBD and periodontitis.

### 4.1. Oral–Gut Dysbiosis: The Translocation of Oral Bacteria

Normally, the human being, by swallowing, ingests large quantities of saliva containing oral bacteria. Many of the bacterial species present in the oral cavity are also found in the intestine of healthy subjects. In the condition of oral and intestinal eubiosis, the bacteria present in the oral cavity are neither capable of infiltrating nor of proliferating at the intestinal level [16,32,57,58]. In conditions of dysbiosis or inflammation (as in the case of IBD), instead, the translocation of bacteria originating from the oral cavity is capable of determining colonization [31,32,33,34,35,36,37]. A study conducted on colonic resections from subjects affected by IBD demonstrated the presence of *Fusobacteria*, *Proteobacteria* and *Firmicutes* in colonic segments with reduced motility compared with adjacent healthy segments [42]. Although many studies agree in stating that periodontopathogen bacteria are capable of significantly influencing the intestinal microbiota by worsening dysbiosis, the study conducted by Miyauchi et al. demonstrated only slight colonization with alteration of bacterial metabolism [38]. The discrepancy in the results could be determined by the small sample sizes and by the lack of standardization of subjects with periodontitis; indeed, only one study analyses patients affected by a specific stage and grade of periodontitis [35]. Further studies are required with homogeneous samples presenting the same stage and grade of disease, identical lifestyle and the same compliance in the treatment of periodontitis, in order to better evaluate the effects of periodontitis on the intestinal microbiota. Murine studies have also been conducted with the aim of evaluating the influence of periodontopathogens on the intestinal microbiota. Specifically, *P. gingivalis* would appear to be capable of colonizing the intestine and of altering the intestinal bacteria [23,24,26,27,28] and fungal [25] composition, worsening dysbiosis [23,24,25,26,27,28]. *A. actinomycetemcomitans* and *F. nucleatum* are also capable of determining an alteration in the intestinal microbial composition [29,30,31]. These studies have made it possible to overcome the bias of sample heterogeneity present in studies conducted on human beings. Conversely, the evaluation of a single strain at a time prevents the assessment of how the various microorganisms are capable of influencing each other in determining the alteration of the intestinal microbiota. Indeed, a study conducted on a murine model demonstrated how *A. actinomycetemcomitans*, if administered together with *S. gordonii*, alters intestinal dysbiosis, the inflammatory response and barrier integrity to a greater extent [29]. In confirmation of what has been stated, the inoculation of saliva from dysbiotic subjects affected by IBD [31] or periodontitis determined an altered inflammatory response and alteration of intestinal barrier integrity [32]. Microbial translocation by pathogens from the oral cavity and the consequent dysbiosis determine the alteration of host–microbiota homeostasis, with inflammatory, metabolic and organ-related consequences. Dysbiosis leads to the replacement of beneficial microorganisms producing SCFAs with pathogenic microorganisms, with consequent alteration of lipid, carbohydrate and amino acid metabolic pathways [23,25,26,36].

### 4.2. Oral–Gut Cross-Talk and Inflammatory Responses

The loss of balance between host and microbiota causes an imbalance between the expression of pro-inflammatory and anti-inflammatory cytokines in favor of the pro-inflammatory ones and the loss of barrier integrity, establishing a vicious cycle that favors the translocation and colonization of oral pathogens [24,26,27,28,29,30,31,32,34,35,36]. The administration of pathogens in a murine model has shown an increase in TNF-α (Tumor Necrosis Factor-α), IL-1β (Interleukin-1β), IFN-γ (Interferon-γ), IL-6 (Interleukin-6) and IRF-1 (Interferon Regulatory Factor 1) [24,26,29,30,59,60]. These results are consistent with those obtained by inoculating saliva from dysbiotic subjects in a murine model [31,32], demonstrating that oral bacteria are capable of worsening intestinal dysbiosis by determining an alteration of the entire intestinal balance of the host and worsening colitis. These microorganisms appear to be capable of acting in synergy, amplifying intestinal damage [29]. These results are consistent with those obtained in subjects affected by periodontitis, which have highlighted, in addition to dysbiosis, the alteration of the inflammatory response [34,35,36] and of intestinal metabolism [36,61]. *P. gingivalis* appears to be capable of increasing intestinal as well as oral inflammation by stimulating the increase in the expression of pro-inflammatory cytokines [24,26,27,28,30] through the activation of Th17 cells [27,28]. The intestine is an organ that induces the production of Th17 cells, whereas the mouth is an organ that receives these cells. Periodontitis could therefore be exacerbated through an immunological link between the intestine and the oral cavity. A study conducted on a murine model has shown that intestinal pre-activation prior to oral infection with *P. gingivalis* determines the accumulation of Th17 cells and an increase in the chemokine CCL20 (Chemokine C-C motif ligand 20), with consequent exacerbation of the pathology. The translocation of the microorganism at the intestinal level and its subsequent absorption at the level of Peyer’s patches have determined a Th17-mediated systemic response [27]. This evidence suggests a strong bidirectional link between mouth and intestine. The two structures are strongly connected and, consequently, the treatment of pathologies affecting one of the two districts requires a treatment that is not strictly limited to the single district but rather a multidisciplinary approach that includes both the mouth and the intestine. The activation and migration of Th17 cells by *P. gingivalis* would appear to be due to the suppression by this bacterium of the linoleic acid pathway [28]. Linoleic acid acts as a ligand of the aryl hydrocarbon receptor (AHR), promoting the differentiation of Treg cells through phosphorylation of STAT1 (Signal Transducer and Activator of Transcription 1) at Ser727 and inhibiting the differentiation of Th17 cells [28]. Consequently, this essential fatty acid could be used as an adjuvant agent in the treatment of both IBD and periodontitis.

The importance of a multi-district approach (mouth and intestine) for the treatment of IBD is supported by the evidence of a reduction in intestinal dysbiosis following non-surgical periodontal treatment [10,11,35,36,37,62,63,64]. The effects of this therapy are not only limited to microbial composition. The treatment of periodontitis would appear to determine not only an improvement in microbiota composition but also a reduction in the inflammatory response and improvement of barrier integrity [35,36,37]. Although most studies are consistent with what has been stated so far, the study by Miyauchi et al. did not find significant beneficial effects of periodontal therapy on the intestinal microbiota [38]. The difference could be due to the different waiting times before reassessment and to the lack of standardization of the sample of subjects affected by periodontitis (only one study analyses periodontal subjects with uniform stage and grade of disease [35]). The different stages and grades of the pathology and, consequently, the different clinical improvements after non-surgical periodontal therapy could determine a different effect at the colonic level. The failure to restore oral eubiosis would determine the maintenance of translocation and subsequent colonization by pathogenic bacteria originating from the oral cavity. Based on the scientific evidence analyzed so far, periodontal therapy could be necessary in order to improve the symptomatology and prognosis of IBD, reducing dysbiosis and thus acting on inflammation [42,45]. The use of probiotics, postbiotics and antibiotics in association with periodontal therapy could further improve IBD outcomes [30,39,40,41].

### 4.3. Modulation of the Oral Microbiome in Patients with IBD

As already stated previously, the relationship between the mouth and the intestine is bidirectional. In the case of IBD, intestinal dysbiosis would appear to negatively influence the oral cavity. In subjects affected by IBD, the presence of oral dysbiosis has been detected [31,43,44,47,48,49,50,51,52,53], characterized by a reduction in alpha diversity and an increase in pro-inflammatory taxa [48,49]. Going into more detail, an increase in anaerobic genera such as *Prevotella* and *Veillonella* [44,51] and a reduction in *Streptococcus*, *Haemophilus* and *Neisseria* [44,54] have been detected. Similarly to what occurs at the intestinal level, the alteration of the oral microbiota in the case of IBD is correlated with an increase in bacterial metabolism of butyrate [49], arginine and isoprene [43], and of inflammation [47]. Although butyrate has beneficial effects at the intestinal level, it would appear to be involved in the progression and onset of periodontitis [4,65]. Also in this district, the alteration of the microbiota caused by IBD can determine an alteration in the structure of oral tissues. In murine models affected by colitis, epithelial hyperplasia has in fact been detected, probably correlated with the reduction in levels of oral *Streptococcus* [46]. Furthermore, an increase in clinical attachment loss has been detected in subjects affected by IBD compared with healthy subjects [47]. Similarly to what has been described at the intestinal level, the treatment of IBD appears to significantly influence the oral cavity. Currently, there are conflicting opinions in the literature regarding the effect of biological drugs on periodontitis. Indeed, two studies in the literature report it: the first, a beneficial effect determined by anti-TNF drugs [55] on the oral microbiota, whereas the second shows a reduction in species richness with an increase in *Prevotella*, *Campylobacter* and *Fusobacterium* in subjects undergoing treatment with biological drugs [54]. The discrepancy in the results could be due to the lack of standardization of the sample. In the first study, subjects undergoing treatment with only anti-TNF are in fact included in the study group, whereas in the second study, subjects undergoing treatment with different biological drugs are included. Further studies are required that analyze more rigorously the effects of biological drugs on the oral cavity. The understanding of the effects of the various drugs used for the treatment of IBD on periodontitis would allow a personalized therapeutic approach in order to maximize therapeutic effects in both districts.

In addition to determining a better therapeutic approach, the analysis of the oral microbiota could allow an easy and non-invasive screening of IBD. Oral pathogens present at the intestinal level would allow the prediction of symptom persistence in subjects affected by Crohn’s disease [45]. Consequently, the identification of specific pathogens at the salivary level could be a valid diagnostic [44,50,53,56], prognostic [45,56], and therapy efficacy assessment aid [55]. Furthermore, the salivary microbiota could be a valid non-invasive aid for the discrimination between Crohn’s disease and Ulcerative Colitis [50,56].

### 4.4. Limitations and Future Perspectives

This scoping review shows the bidirectional correlation between periodontitis and IBD. Although the link between the mouth and the gut has been highlighted from a microbial, immunological, and molecular point of view, limitations are present. The studies examined exhibit great heterogeneity in study design and the method utilized, which limits the level of evidence. Works conducted on animal models focusing on a single microorganism are unable to reproduce salivary polymicrobial pathogenicity. Moreover, the use of small sample sizes and the lack of standardized criteria for assessing disease status, which would allow evaluation of how disease severity may influence the results, further limit the level of evidence. A further limitation is given by the lack of long-term studies that allow for an evaluation of how disease evolution and therapeutic protocols influence microbiota, as well as the inflammatory and metabolic status in the two districts. Indeed, the high heterogeneity of the included studies regarding sample size, disease staging, and duration of follow-up, in the absence of a quantitative meta-analysis. Finally, marker salivary biomarkers exclusively identify an association and not a cause–effect relationship, suffering from the lack of both predictive algorithms robustly validated on a large scale. The interpretation of our results is therefore limited by the methodological criticalities found in the selected studies, particularly the intrinsic heterogeneity of the included literature, together with the presence of uncontrolled confounding biases within the primary studies.

Awareness of this bidirectional connection between the mouth and the gut lays the foundations for a multidisciplinary approach to the management of periodontitis and IBD. Therapy should not be limited to the treatment of the specific district but should be associated with periodic monitoring of the health status of both districts and eventual treatment in case of pathology. This new multispecialty approach would improve the state of the disease, reducing progression and flare-ups, and improving the response to therapy.

Furthermore, the correlation between these two districts highlights the possibility of using salivary markers for the monitoring of IBD. This new methodology would allow for a simple and non-invasive screening of all subjects with risk factors, more frequent monitoring of affected subjects, and the early identification of subjects at risk of flare-ups or lack of response to therapy.

Long-term studies on large populations are required to evaluate microbial, immunological, and molecular variations, taking into account subjects with Crohn’s disease, with Ulcerative Colitis, with periodontitis, and with combinations of them compared with healthy subjects, evaluating how the stage of the disease and therapy influence the markers.

## 5. Conclusions

Given scientific evidence regarding the oral–gut axis, this scoping review highlights the bidirectionality of this relationship and how it can be exploited for the management of periodontitis and IBD. The management of these two pathologies must not be reduced to an action limited to the single affected district but must be carried out with a multidisciplinary approach. Going into detail, the restoration of intestinal eubiosis could be an adjuvant treatment to periodontal therapy capable of determining an improvement in outcomes and a lower risk of recurrence. Conversely, the restoration of oral eubiosis could be capable of reducing the risk of progression and flare-ups of IBD. Furthermore, salivary analysis could allow for a less invasive approach for diagnosis, prognostic evaluation, and response to therapy in cases of IBD. Given the high heterogeneity of the results, further studies on larger populations are necessary to standardize the findings and identify a specific molecular and/or microbial signature. These studies lay the foundations for a new multidisciplinary approach and for personalized medicine that aims for the correct management of periodontitis and IBD.

## Figures and Tables

**Table 1:** Characteristics and outcomes of most relevant included studies of this review.

Author	Aim of the Study	Study Design	Sample	Outcome	Main Findings
Kato et al. (2018) [23]	To investigate the effects of *P. gingivalis* on gut microbiota	In vivo animal study	*P. gingivalis: n* = 7 Control: *n* = 7	Gut microbiota composition, predicted metabolic pathways	Gut dysbiosis and systemic metabolic alterations. *P. gingivalis*-induced dysbiosis may contribute to systemic diseases by altering host–microbiota homeostasis.
Schmidt et al., (2018) [47]	To investigate the relationship between aMMP-8, periodontal pathogens, and IBD	Observational cross-sectional study	CD: *n* = 29 UC: *n* = 30 HC: *n* = 59	aMMP-8 levels (GCF), periodontal parameters, periodontal pathogens	IBD patients: ↑ CAL, ↑ PBI, ↑ aMMP-8 (GCF) ↓ *Eubacterium nodatum* in IBD; ↓ *Eikenella corrodens* overall but ↑ in CD vs. UC.
Xun et al. (2018) [56]	To characterize the salivary microbiota in IBD	Observational case–control study with cross-sectional analysis	UC: *n* = 54 CD: *n* = 13 HC: *n* = 25	Identification potential microbial biomarkers in IBD	IBD: dysbiotic salivary microbiota. *Corynebacterium*, *Acinetobacter*, *Lactobacillus*, *Bifidobacterium*, *Streptococcus*, *Pseudomonas* were identified as disease-specific indicators.
Lourenço et al., (2018) [33]	To evaluate gut microbiome alterations in periodontitis.	Cross-sectional observational study with metagenomic analysis	Periodontitis: *n* = 23 Gingivitis: *n* = 14 HC: *n* = 7	Gut microbiota composition and association with periodontal status.	Periodontitis: ↑ *Firmicutes*/*Bacteroidetes* ratio and ↑ *Euryarchaeota*, *Verrucomicrobia*, and *Proteobacteria.* Evidence of oral–gut translocation.
Dinakaran et al. (2019) [42]	To evaluate the interaction between oral and gut microbiome in colitis	Observational cross-sectional study	CD: *n* = 13 UC: *n* = 13 HC: *n* = 26	Microbiome analysis on diseased colon tissue and adjacent healthy colonic tissue from the same patients	Colitis samples: significant dysbiosis with a predominance of pathogenic oral bacteria suggesting oral–gut translocation. ↑ *Firmicutes*, *Fusobacteria* and *Proteobacteria* associated with inflammation.
Gatej et al. (2020) [30]	To investigate the effects of *P. gingivalis* and *F. nucleatum* on gut microbiota and the effect of *Lactobacillus Rhamnosus GG*	In vivo animal study	PD: *n* = 6 LGG oral: *n* = 6 LGG gav: *n* = 6 PD + LGG oral: *n* = 6 PD + LGG gav: *n* = 6	IL-6 expression, microbiome analysis	*P. gingivalis* and *F. nucleatum*: ↑ IL-6, loss of *Clostridiales* taxa (dysbiosis). LGG: restored beneficial taxa such as *Lachnospiraceae* (*Clostridiales*) and reduced taxa associated with disease; ↓ IL-6.
Qian et al. (2022) [32]	To investigate whether periodontitis-derived salivary microbiota can exacerbate DSS-induced colitis	In vivo animal study	HC: *n* = 9 Periodontitis: *n* = 9 DSS-treated: *n* = 9 DSS + periodontitis: *n* =9	Disease severity, cytokines, gut microbiota, metabolomics	Dysbiotic salivary microbiota ↑ disease activity index, ↑ histological damage, ↑intestinal inflammation and ↓ intestinal barrier integrity.
Tsuzuno et al. (2021) [24]	To investigate oral ingestion of *P. gingivalis*, *P. intermedia*, *F. nucleatum* affect colitis	In vivo animal study	P. g: *n* = 6 P. i: *n* = 6 F. n: *n* = 6 Control: *n* = 6	Disease activity index, inflammatory cytokines, intestinal barrier proteins	*P. gingivalis* drives inflammation, dysbiosis, and barrier dysfunction; *P. intermedia* enhances inflammatory responses, supporting a role in the oral–gut axis.
Simas et al. (2021) [41]	To investigate the effects of periodontitis on systemic inflammation and gut microbiota	In vivo animal study	Baseline: *n* = 6 Antibiotic (A): *n* = 6 Vehicle (V): *n* = 7 P. g: *n* = 8 V + A: *n* = 7 P. g + A: *n* = 8	Gut microbiota composition, intestinal inflammation, cytokine expression, alveolar bone loss	Periodontitis contributed to dysbiosis, intestinal barrier dysfunction and increased permeability. Treatment intervention partially restored microbial balance and reduced inflammatory markers, improving gut integrity and reducing systemic inflammation.
Park et al. (2021) [39]	To evaluate the effects of postbiotics derived from *E. faecalis* and *L. lactis* on periodontitis and gut inflammation	In vivo animal study	PD: *n* = 8 PD + E. f: *n* = 8 PD + E. F + L. l: *n* = 8 Control: *n* = 8	Periodontal parameters, cytokine expression, gut microbiota composition	*E. faecalis* and *E. faecalis* + *L. lactis*: ↓ periodontal inflammation and bone loss. Periodontal tissue: TNF-α ↓, IL-1β ↓, IL-6 ↓, GPx1 ↑, SOD1 ↑. Gut: TNF-α ↓, IL-1β ↓, ↑ IL-10, ↑ SOD1. Gut microbiota: ↑ *Lactobacillus* spp. Postbiotics improved both periodontal and gut health.
Choi et al. (2021) [40]	To evaluate the effects of *L. curvatus* SMFM2016-NK- fermented milk on gut microbiota	In vivo animal study	Treatment: *n* = 8 Normal, Negative, Positive control: *n* = 8	Gene expression of pro-inflammatory cytokines and gut	Gut: ↓ α-diversity. Combined anti-inflammatory and antimicrobial effect, reducing pathogenic bacteria in intestinal environments.
Imai et al. (2021) [48]	To investigate the relationship between oral and gut microbiota in patients with IBD	Observational cross-sectional study	IBD Group: *n* = 60 HC: *n* = 45	Oral and gut microbiota composition and identification of IgA-coated bacteria associated with IBD	IBD: oral and gut dysbiosis, with specific enrichment of IgA-coated bacteria. IgA-targeted microbes were more prevalent in disease conditions, indicating immune recognition of pro-inflammatory taxa.
Hu et al., (2021) [43]	To investigate the relationship between oral and gut microbiome in Crohn’s disease	Observational cross-sectional study	CD: *n* = 25 HC: *n* = 25	Salivary and gut microbiome (stool) analyzed via meta-genomics	Gut colonization by oral bacteria. Salivary ↑ *Streptococcus* spp. and ↓ *Bacteroides* spp. in CD vs. HC Enrichment of arginine biosynthesis and isoprene pathways associated with inflammation.
Chen et al. (2022) [25]	To evaluate the effect of *P. gingivalis* on gut mycobiome	In vivo animal study	*P. gingivalis* group vs. Control group	Analysis of gut mycobiome, bacteria–fungi interactions, metabolic pathways (KEGG).	*P. gingivalis* alters gut mycobiome composition. Mycobiome changes correlated with lipid metabolites and decreased tryptophan metabolites.
Liu et al. (2022) [26]	To investigate the effects of *P. gingivalis* on gut	In vivo animal study	*P. gingivalis* vs. PBS (control)	Gut microbiota composition, immune activation, and inflammation	Significant dysbiosis of the gut microbiota (↓ bacterial diversity and ↑ *Bacteroidetes*/*Firmicutes ratio*). ↓ in beneficial SCFA-producing taxa, ↑ TNF-α, IFN-γ, and IRF-1.
Park et al. (2022) [55]	To evaluate whether microbiome profiles can predict response to anti-TNF-α therapy in patients with IBD	Observational study	IBD: *n* = 19 Healthy control: *n* = 19	Differences in microbiome composition associated with treatment response	Responders: ↑ beneficial taxa in feces and saliva. Multi-site microbiome profiling demonstrated potential as a predictive biomarker for anti-TNF-α therapy response in IBD patients.
Abdelbary et al. (2022) [44]	To characterize salivary and gut microbiota in IBD patients and healthy controls	Observational case–control study	IBD: *n* = 14 HC: *n* = 12	Microbial composition, diversity, taxa abundance	IBD: salivary dysbiosis characterized by ↑ *Prevotella* and *Veillonella* and ↓ *Streptococcus*, *Haemophilus*, and *Neisseria*. Oral microbiota may contribute to IBD pathogenesis and serve as potential biomarkers.
Nagao et al. (2022) [27]	To investigate how *P. gingivalis* influences intestinal microbioma, immunity and inflammation	In vivo animal study	Intestine: *n* = 9 Oral: *n* = 9 Intestine+/oral+: *n* = 9 Control: *n* = 7	Intestinal immune response, microbiota alterations, inflammatory markers	*P. gingivalis* or its OMVs induces alterations in overall microbial composition. *↑ IL-17A*, *↑ RORγt*, and *↑ CCR6*, indicating activation and migration potential of Th17 cells.
Hu et al. (2022) [49]	To evaluate the role of the oral microbiome in the diagnosis and oral manifestations of CD	Observational cross-sectional study	CD with oral le-sions: *n* = 16 CD without oral lesions: *n* = 25 HC: *n* = 24	Differences in oral microbiome composition	Oral dysbiosis on CD. ↑ pro-inflammatory taxa, ↓ protective species. Evidence of ectopic colonization of gut-associated bacteria in the oral cavity. ↑ butyrate production.
Kang et al. (2023) [50]	To identify oral microbiome patterns in IBD and evaluate microbial biomarkers using machine learning	Prospective clinical study	UC: *n* = 175 CD: *n* = 127 HC: *n* = 100	Diagnostic performance of ML models (sPLS-DA) and oral microbiome diversity	Oral microbiome diversity ↓in IBD. Machine learning models showed high accuracy in distinguishing IBD vs. controls and good performance for CD vs. UC. The oral microbiome may serve as a non-invasive diagnostic biomarker.
Hammad et al. (2023) [51]	To evaluate oral microbial on IBD	Combined clinical and in vitro experimental study	IBD: *n* = 14 HC: *n* = 12	Characterization of oral microbial abundance and species distribution	IBD: *↑ Veillonella* spp., *Prevotella* spp. (specifically *Prevotella salivae*). Presence of a dysbiotic oral microbiota in IBD characterized by enrichment of anaerobic genera.
Jia et al. (2024) [28]	To investigate the effect of *P. gingivalis* on colitis and to elucidate the underlying gut microbiota–metabolite–immune mechanisms	In vivo animal study	WT P. g: *n* = 5 WT P. g + LA: *n* = 5 KO P. g: *n* = 5 KO P. G + LA: *n* = 5	Assessment of colitis, gut microbiota composition, metabolite profiling, immune response	*P. gingivalis* aggravated DSS-induced colitis. LA supplementation reversed these effects by restoring AHR signaling, rebalancing Th17/Treg cells, and significantly ameliorating colitis severity. *P. gingivalis* promotes colitis through a gut microbiota–LA–AHR–Th17/Treg axis.
Rocha et al. (2024) [29]	To evaluate the effects of oral infection with *A. actinomycetemcomitans* and/or *S. gordonii* on gut	In vivo animal study	Negative Control: *n* = 12 Aa Group: *n* = 12 Sg Group: *n* = 12 Aa + Sg Group: *n* = 12	Alveolar bone loss; oral and gut microbiota; cytokine gene ex-pression; intestinal barrier genes	*A. actinomycetemcomitans* + *S. gordonii*: Oral: enhanced dysbiosis compared with single infection; Gut: more pronounced dysbiosis vs. single groups; ↑Cytokines pro-inflammatory; stronger dysregulation of tight junctions and mucins; Synergistic role of *A. actinomycetemcomitans* and *S. gordonii* in the oral–gut axis.
Baima et al. (2024) [37]	To evaluate the effect of periodontitis and its non-surgical treatment on oral and gut micro-biota composition	Prospective clinical study	Stage III/IV periodontitis: *n* = 47 HC: *n* = 47	Changes in oral and gut microbiota before and after therapy	PD: dysbiotic oral and gut microbiota. ↑ pro-inflammatory taxa. NSPT: partial restoration of microbial balance, with limited but notable modulation of gut microbiota.
DeClercq et al. (2025) [52]	To evaluate differences in the salivary oral microbiome in IBD	Observational cross-sectional study	IBD group: *n* = 160 HC: *n* = 160	Analysis of salivary microbiome	↓ alpha diversity in IBD patients. Beta diversity differences indicate altered microbial composition in CD vs. controls, while UC shows more similar profiles to controls.
Golob et al. (2024) [45]	To evaluate gut microbiome alterations in patients with quiescent CD with persistent symptoms	Multicenter observational study with metagenomic analysis	qCD+S: *n* = 39 qCD-S: *n* = 274 aCD: *n* = 21 HC: *n* = 50 IBS: *n* = 40	Gut microbiome and association with symptoms	qCD+S: ↑ oral-associated bacteria and ↓ butyrate- and indole-producing species. The microbiome can predict persistent symptoms.
Wang et al. (2025) [46]	To investigate whether chronic colitis induces oral microbiota dysbiosis and oral mucosal alterations	In vivo animal study	DSS-induced chronic colitis: *n* = 15 Control: *n* = 15	Oral microbiota composition, epithelial thickness	Chronic colitis induced oral epithelial hyperplasia. Chronic colitis drives oral dysbiosis characterized primarily by depletion of *Streptococcus*, which may contribute to epithelial hyperplasia.
Yuan et al. (2025) [53]	To develop and validate a metaproteomic workflow (OSaMPle) for oral microbiome analysis and to investigate functional alterations of the oral microbiome in IBD	Observational clinical study with metaproteomic analysis	GX: *n* = 3 UX: *n* = 3 GT: *n* = 3 UT: *n* = 3 Control group (*n* = 3)	Differences in oral microbiome protein expression between IBD and healthy subjects	IBD: the oral microbiome showed functional dysbiosis rather than only taxonomic shifts. OSaMPle workflow significantly improves microbial protein detection by reducing host protein interference.
Miyauchi et al. (2025) [38]	To investigate the oral–gut axis in periodontitis	Longitudinal observational clinical study	PD: *n* = 27 HC: *n* = 27	Gut and salivary, metabolomic profiles	Gut: Minimal direct ectopic colonization. Alterations in microbial metabolic activity. Periodontal treatment: minimal impact on gut microbiota.
Wark et al. (2025) [54]	To investigate the relationship between oral microbiome and biologic therapy	Observational cross-sectional study	IBD: *n* = 57 HC: *n* = 24	Oral microbiome and association with biologic therapy	↑ *Haemophilus* in healthy controls and ↓ IBD. Patients on biologics: ↓ species richness; ↑ *Prevotella*, *↑ Campylobacter*, *↑ Fusobacterium* Oral microbial diversity is influenced by biologic treatment.
Paz et al. (2026) [34]	To evaluate familial patterns of oral–gut dysbiosis in periodontitis patients	Observational cross-sectional study	PD parents: *n* = 20 Healthy parents: *n* = 20 PD children *n* = 20 Healty children: *n* = 20	Oral (saliva) and gut microbiome, immune markers and clinical periodontal parameters	Gut dysbiosis with ↑ pro-inflammatory bacteria and oral taxa Familial patterns: partially shared oral and gut microbiota ↓ IFN-γ and altered IL-10 response.
Baima et al., 2025 [36]	To evaluate the effect of NSPT on oral and gut microbiome and metabolome	Longitudinal observational study	PD: *n* = 25 HC *n* = 25	Oral (saliva) and gut microbiome, metabolomics and microbial functional pathways	Gut dysbiosis and oral-to-gut bacterial translocation ↓ SCFAs, amino acids, sugars; ↑ pro-inflammatory metabolites NSPT partially restores oral and gut microbiota.
Mutafcilar Velioglu et al. (2025) [35]	To evaluate the effect of NSPT on oral and gut microbiota in PD	Prospective clinical study with metagenomic analysis	PD: *n* = 5 HC: *n* = 5	Periodontal clinical parameters and salivary and gut microbiota composition	Gut: ↓ *Firmicutes*/*Bacteroidetes* ratio. Oral: ↓ pathogenic phyla, ↑ *Firmicutes*. Changes in gut microbiota correlates with clinical improvements.
Chen et al. (2026) [31]	To investigate the relationship between oral microbiota dysbiosis and intestinal inflammation	Experimental animal study	Mice (4 groups *n* = 5); Oral samples: IBD: *n* = 28 Control: *n* = 21	Evaluation of colitis severity, immune response, and microbiota composition	Oral microbiota dysbiosis ↑ intestinal inflammation, ↓ intestinal barrier integrity, with specific periodontal pathogens promoting colitis.

Legend: P.g: *Porphyromonas gingivalis*; P.i: *Prevotella intermedia*; F.n: *Fusobacterium nucleatum*; E.e: *Enterococcus faecalis*; Ll: *Lactococcus lactis*; LGG: *Lactobacillus rhamnosus GG* Aa: *Aggregatibacter actinomycetemcomitans*; S.g: *Streptococcus gordonii*; aMMP-8: active MMP-8; PD: periodontitis; GCF: gingival crevicular fluid; CAL: clinical attachment level; PBI: papillary Bleeding Index; HC: Healthy control; CD: Chron’s Disease; UC: Ulcerative Colitis; LA: linoleic acid; NSPT: Non-surgical periodontal therapy; qCD+S: quiescent CD with symptoms; qCD-S: quiescent CD without symptoms; aCD: active CD; IBS: Irritable Bowel Syndrome; AHR: Aryl Hydrocarbon Receptor; IL-10: Interleukin-10; IL-1β: Interleukin-1β; IL-6: Interleukin-6; TNF-α: Tumor Necrosis Factor-α; GPx1: Glutathione Peroxidase 1; SOD1: Superoxide Dismutase; IFN-γ: Interferon-γ; IRF-1: Interferon Regulatory Factor 1; IL-17A: Interleukin-17A; RORγt: Retinoic-related Orphan Receptor-γt; CCR6: C-C chemokine receptor type 6; GX: samples resuspended in GX buffer containing 4 M GuHCl and 0.5% Triton X-100 in PBS; UX: samples resuspended in UX buffer containing 6 M urea and 0.5% Triton-X 100 in PBS; GT: Samples resuspended in GT buffer containing 4 M GuHCl and 0.5% Tween 80 in PBS; UT: Samples resuspended in UT buffer containing 6 M urea and 0.5% Tween 80 in PBS.

## Data Availability

Data are available from the corresponding author upon reasonable request.
